# Systematic Review and Meta-Analysis of the Efficacy of Fecal Microbiota Transplantation in Parkinson’s Disease: An Exploration Based on UPDRS and Cognitive Scores

**DOI:** 10.31083/RN50106

**Published:** 2026-06-26

**Authors:** Benyao Liang, Jie Zou, Xiangyu Mao, Ning Xie, Zhaohui Liang

**Affiliations:** ^1^Department of Neurology, Loudi Central Hospital, 417000 Loudi, Hunan, China

**Keywords:** fecal microbiota transplantation, gut-brain axis, meta-analysis, Parkinson’s disease, randomized controlled trial

## Abstract

**Background:**

Parkinson’s disease (PD) is a common neurodegenerative disorder that has been increasingly linked to gut-brain axis dysfunction. Fecal microbiota transplantation (FMT), a microbiome-targeted intervention, has shown theoretical and preliminary clinical potential in PD, but randomized clinical evidence remains limited. This review aimed to systematically evaluate the effects of FMT on motor, non-motor, and cognitive outcomes in patients with PD.

**Methods:**

A Preferred Reporting Items for Systematic Reviews and Meta-Analyses (PRISMA)-compliant systematic review and meta-analysis of randomized controlled trials (RCTs) comparing FMT with placebo or conventional care in PD was conducted. Two reviewers independently screened studies, extracted data, and assessed risk of bias. Pooled analyses were performed using a random-effects model, and the certainty of evidence was evaluated using the Grading of Recommendations, Assessment, Development, and Evaluation (GRADE) approach.

**Results:**

Five RCTs involving 226 participants were included. No statistically significant differences were observed between the FMT and control groups in Movement Disorder Society Unified Parkinson’s Disease Rating Scale (MDS-UPDRS) Parts I–III, Montreal Cognitive Assessment (MoCA), or Mini-Mental State Examination (MMSE) scores at any assessed follow-up time, with heterogeneity generally low to moderate across outcomes.

**Conclusions:**

Based on current evidence from five small RCTs, FMT did not demonstrate a statistically significant benefit for motor, daily living, or cognitive outcomes in PD. However, these findings should be interpreted cautiously, given the limited sample size, short follow-up duration, and between-study differences in intervention protocols. Larger, well-designed RCTs with standardized FMT protocols and longer follow-up are needed.

**The PROSPERO Registration:**

This systematic review was registered in the PROSPERO database under registration number CRD420251121443, https://www.crd.york.ac.uk/PROSPERO/view/CRD420251121443.

## 1. Introduction

Parkinson’s disease (PD) is the second most common neurodegenerative disorder worldwide and represents an increasingly prevalent source of disability and reduced quality of life in older adults [1,2,3]. In addition to its characteristic motor manifestations, PD is frequently accompanied by gastrointestinal dysfunction and a broad range of non-motor symptoms. Growing evidence suggests that the gut-brain axis may play an important role in PD pathogenesis, and alterations in gut microbiota composition have been linked to intestinal barrier dysfunction, inflammation, and abnormal neuroimmune signaling [4,5,6].

Gut microbiota dysbiosis has therefore emerged as a potential therapeutic target in PD. Previous studies have shown that patients with PD exhibited altered microbial composition, including reduced abundance of beneficial bacteria such as *Faecalibacterium prausnitzii*, together with relative enrichment of pro-inflammatory taxa [7,8,9,10]. Experimental evidence has further suggested that PD-associated microbiota may contribute to motor and neuropathological abnormalities [9,10]. Because gut microbiota may influence short-chain fatty acid (SCFA) production, intestinal permeability, and inflammatory signaling, interventions aimed at restoring microbial balance have attracted increasing clinical interest. Among these, fecal microbiota transplantation (FMT) has emerged as one of the most direct approaches to modulating the microbiota.

FMT has shown therapeutic value in several gastrointestinal and metabolic conditions [11,12,13,14]. In PD, early clinical studies suggested that FMT may reduce constipation and some motor or non-motor symptoms in certain patients [15,16,17]. However, the available clinical evidence remains limited, and findings across studies have been inconsistent. In addition, differences in fecal preparation, route of administration, dosing frequency, and follow-up duration may have further complicated the interpretation of treatment effects.

Given the emerging but inconclusive evidence base, a systematic synthesis of randomized controlled trials (RCTs) was needed. Therefore, the present systematic review and meta-analysis synthesized RCT evidence on the effects of FMT on motor, non-motor, daily living, and cognitive outcomes in patients with PD.

## 2. Methods

### 2.1 Data Sources and Search Strategy

This systematic review and meta-analysis were conducted in accordance with the Preferred Reporting Items for Systematic Reviews and Meta-Analyses (PRISMA) statement and were prospectively registered in the PROSPERO database (registration number: CRD420251121443). A comprehensive literature search was performed from database inception to September 15, 2025, in PubMed (https://pubmed.ncbi.nlm.nih.gov/), Ovid Embase (https://www.embase.com/), Scopus (https://www.scopus.com/), and the Cochrane Central Register of Controlled Trials (CENTRAL) (https://www.cochranelibrary.com). Ongoing or unpublished trials were identified solely via the above databases, and ClinicalTrials.gov (https://clinicaltrials.gov/) was not searched. The reference lists of all eligible articles and relevant reviews were also screened manually to identify additional studies. The search strategy combined controlled vocabulary terms and free-text keywords such as “Parkinson’s disease”, “fecal microbiota transplantation”, and “randomized controlled trials”, with database-specific adaptations as appropriate. The full search strategies for each database are provided in the **Supplementary Material**.

### 2.2 Eligibility Criteria

The eligibility criteria were defined according to the Population, Intervention, Comparator, Outcome, Study design (PICOS) framework.

Sample: Patients with a clinical diagnosis of Parkinson’s disease.

Intervention: FMT, regardless of fecal preparation type, route of administration, or dosing frequency.

Comparator*:* Placebo, sham treatment, conventional care, or other control interventions.

Outcomes: At least one relevant motor, non-motor, or cognitive outcome reported quantitatively, including Movement Disorder Society Unified Parkinson’s Disease Rating Scale (MDS-UPDRS)/UPDRS scores, Montreal Cognitive Assessment (MoCA), Mini-Mental State Examination (MMSE), or related clinical measures.

Study design: RCTs.

Studies were excluded if they were non-randomized studies, observational studies, case reports, case series, reviews, conference abstracts, trial protocols, or other unpublished reports without extractable outcome data.

### 2.3 Study Selection

**Fig. 1.**
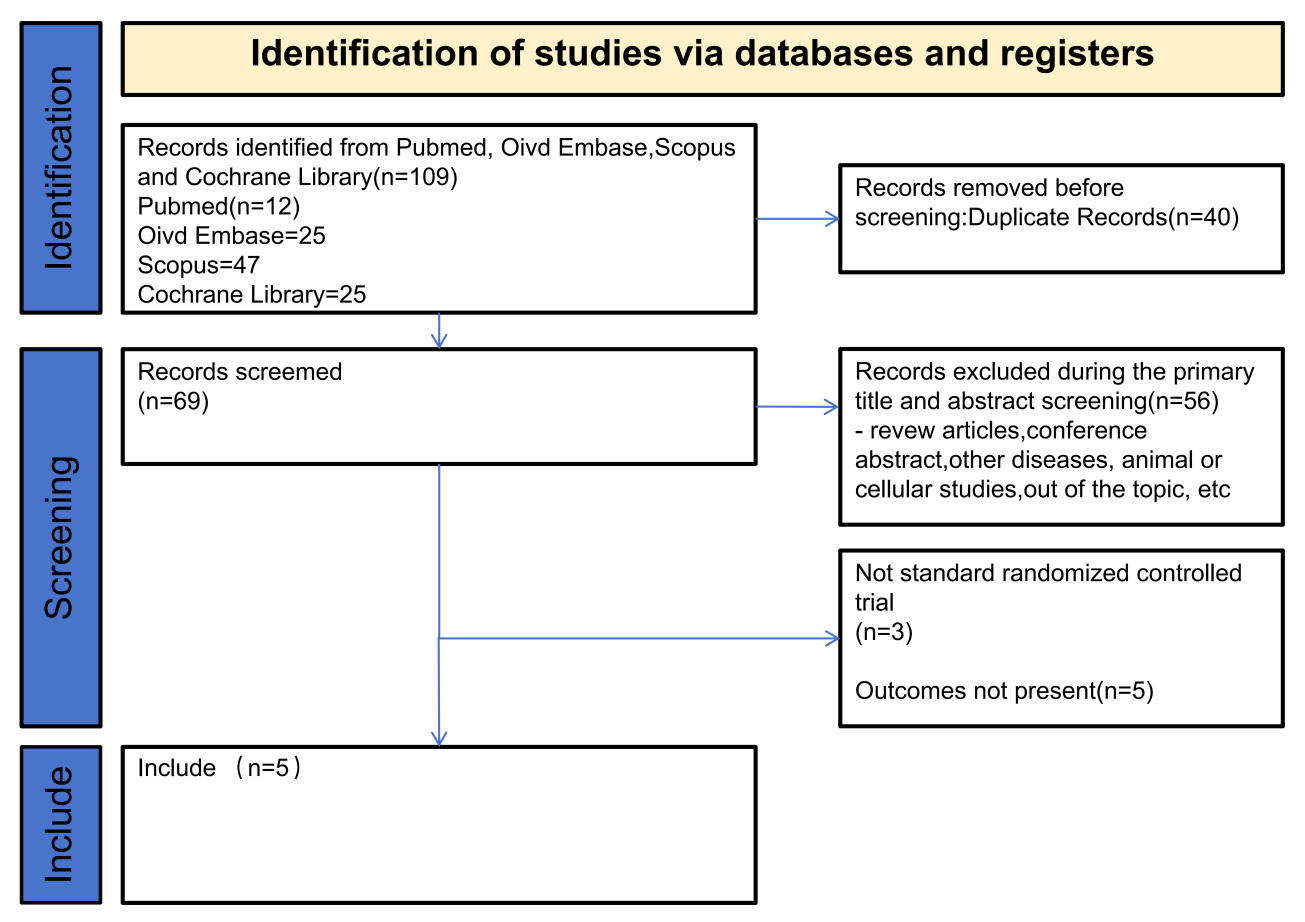
**PRISMA flowchart of the study selection process**. PRISMA, Preferred Reporting Items for Systematic Reviews and Meta-Analyses.

All retrieved records were imported into EndNote 21 (Clarivate Analytics, Philadelphia, PA, USA) for reference management and duplicate removal. Study selection was performed in two stages: first, titles and abstracts were screened; second, the full texts of potentially eligible articles were assessed in detail. Two reviewers independently performed study screening. Any disagreements were resolved through discussion, and when necessary, through consultation with a third reviewer. The study selection process is summarized in the PRISMA flow diagram in Fig. 1.

### 2.4 Data Extraction and Quality Assessment

Two reviewers independently extracted data using a standardized data extraction form in Microsoft Excel. Discrepancies were resolved through discussion with a third reviewer. The following information was collected from each included study: study identifier, country, study design, sample size, participant baseline characteristics, details of the FMT intervention and control condition, follow-up duration, and outcome data. The primary outcomes were changes in MDS-UPDRS Parts I–III at prespecified follow-up time points. Cognitive outcomes, including MoCA and MMSE scores, were analyzed as secondary outcomes when available. Risk of bias was assessed independently by two reviewers using the Cochrane Risk of Bias tool for randomized trials. The following domains were evaluated: randomization process, deviations from intended interventions, missing outcome data, measurement of outcomes, and selection of the reported result. Any disagreements were resolved by discussion with a third reviewer. The certainty of evidence for each outcome was evaluated independently by two reviewers using the Grading of Recommendations, Assessment, Development, and Evaluation (GRADE) approach, considering risk of bias, inconsistency, indirectness, imprecision, and publication bias. Reasons for downgrading the certainty of evidence are presented in Table 1.

**Table 1.** **GRADE summary of findings**.

Outcomes	Anticipated absolute effects (95% CI)	Mean difference (95% CI)	n of participants (studies)	Certainty of the evidence (GRADE)
	Risk with placebo	Risk with FMT		
Change in MDS-UPDRS Part I (week 12)	The mean change in MDS-UPDRS I ranged from –2.74 to 0.36	MD –0.91 lower (2.22 lower to 0.39 higher)	130 (3 RCTs)	Moderate^a^
Change in MDS-UPDRS Part I (week 24)	The mean change in MDS-UPDRS I ranged from –2.2 to –0.08	MD 0.70 higher (–0.96 lower to 2.35 higher)	91 (2 RCTs)	Moderate^a^
Change in MDS-UPDRS Part II (week 12)	The mean change in MDS-UPDRS II ranged from –4.74 to 0.5	MD –1.83 lower (3.69 lower to 0.03 higher)	130 (3 RCTs)	Moderate^a^
Change in MDS-UPDRS Part II (week 24)	The mean change in MDS-UPDRS II ranged from –0.27 to 1.3	MD 0.09 higher (–1.65 lower to 1.84 higher)	91 (2 RCTs)	Moderate^a^
Change in MDS-UPDRS Part III (week 4)	The mean change in MDS-UPDRS III ranged from –8.53 to –0.61	MD –2.42 lower (5.97 lower to 1.12 higher)	135 (3 RCTs)	Moderate^a^
Change in MDS-UPDRS Part III (week 12)	The mean change in MDS-UPDRS III ranged from –8.86 to –0.07	MD –2.79 lower (9.04 lower to 3.47 higher)	130 (3 RCTs)	Low^a,b^
Change in MDS-UPDRS Part III (week 24)	The mean change in MDS-UPDRS III ranged from –5.6 to –1.5	MD 1.65 higher (–1.84 lower to 5.13 higher)	91 (2 RCTs)	Moderate^a^
Change in MoCA Score (week 4)	The mean change in MoCA ranged from –1.46 to 3.89	MD 0.16 higher (–1.01 lower to 1.32 higher)	135 (3 RCTs)	Moderate^a^
Change in MMSE Score (week 4)	The mean change in MMSE ranged from –0.56 to 1.89	MD 0.79 higher (–0.36 lower to 1.94 higher)	135 (3 RCTs)	Moderate^a^

Note: CI, confidence interval; MD, mean difference; RCT, randomized controlled trial; FMT, fecal microbiota transplantation; MDS-UPDRS, Movement Disorder Society Unified Parkinson’s Disease Rating Scale; MoCA, Montreal Cognitive Assessment; MMSE, Mini-Mental State Examination; GRADE, Grading of Recommendations, Assessment, Development, and Evaluation; ^a^ 95% Confidence Interval (CI) is wide and crossing 0; ^b^ Heterogeneity (I^2^) >50% (as specified in each outcome).

### 2.5 Data Synthesis and Statistical Analysis

For continuous outcomes, pooled-effect estimates were expressed as mean differences (MDs) with 95% confidence intervals (CIs). Meta-analyses were performed using Review Manager software (RevMan, version 5.4.1, The Cochrane Collaboration, London, UK). Statistical heterogeneity was assessed using the I² statistic. A fixed-effect model was used when heterogeneity was low or negligible, while a random-effects model was applied when substantial heterogeneity was observed, generally defined as I² > 50%.

When change-from-baseline SDs were not directly reported, they were calculated using the following formula: $SD_{\text{change}}=\sqrt{{\left(SD_{\text{pre}}\right)}^{2}+{\left(SD_{\text{post}}\right)}^{2}-\left(2r\times SD_{\text{pre}}\times SD_{\text{post}}\right)}$ [18], where r represents the correlation coefficient between baseline and follow-up measurements. Because this value was not reported in the original studies, r was assumed to be 0.5 for the primary analysis. Sensitivity analyses using r = 0.3 and r = 0.7 were additionally performed, and the pooled effect estimates remained materially unchanged, indicating that the findings were robust to different assumptions regarding the correlation coefficient.

Additionally, when studies reported medians instead of means, median values were converted to means using the method described by Wan et al. [19]. A total of 2 of the 5 included studies required median-to-mean conversion. Because such conversion may have introduced bias, particularly in small samples or skewed data distributions, this limitation was considered during the interpretation of the results.

Statistical heterogeneity was assessed using the Cochran Q test and the Higgins I^2^ statistic. An I^2^ value greater than 50% was considered indicative of substantial heterogeneity. When substantial heterogeneity was detected, sensitivity analysis using a leave-one-out approach was performed to explore potential sources of heterogeneity [20].

Publication bias was explored using funnel plots. However, because fewer than 10 studies were included in each meta-analysis, the interpretability of funnel plot symmetry was limited, and publication bias could not be reliably assessed.

## 3. Results

### 3.1 Literature Selection

A comprehensive search initially identified 109 articles. After removal of 40 duplicate entries, we retained a total of 69 unique articles for further evaluation. Subsequent screening based on titles and abstracts excluded another 56 articles. Eventually, according to the preset inclusion criteria, 13 studies that met the full-text assessment conditions were retained. Ultimately, this meta-analysis encompassed five RCTs (Fig. 1).

### 3.2 Study Characteristics

The present meta-analysis included five randomized controlled trials that met the predefined eligibility criteria. Among these studies, one was conducted in Belgium, one in Finland, and three in China. The included trials were published between 2023 and 2025. In total, 226 participants were included, of whom 119 received FMT and 107 received placebo or conventional care. Across studies, the mean or median age of participants ranged from 60.5 to 73.8 years, body mass index (BMI) ranged from 22.3 to 26.2, and PD duration ranged from 3.0 to 7.0 years. The characteristics of the included studies and participants are summarized in Table 2 (Ref. [21,22,23,24,25]).

**Table 2.** **Characteristics of the included studies and participants**.

Study	Location	Study design	Sample size		Age (years)		BMI (kg/m^2^)		PD duration (years)	
			FMT	Placebo	FMT	Placebo	FMT	Placebo	FMT	Placebo
Bruggeman et al., 2024 [21]	Belgium	RCT	22	24	61.0	60.5	24.6	24.3	4.2	4.4
Scheperjans et al., 2024 [22]	Finland	RCT	30	15	66.0^a^	65.0^a^	26.2^a^	26.1^a^	5.9^a^	7.0^a^
Liu et al., 2023 [23]	China	RCT	25	26	70.9	73.8	N/A	N/A	3.4	3.9
Wang et al., 2025 [24]	China	RCT	15	15	67.0^a^	68.0^a^	24.9	25.4	3.0^a^	3.0^a^
Cheng et al., 2023 [25]	China	RCT	27	27	60.5	62.6	22.3	22.4	6.7	5.9

Note: PD, Parkinson’s disease; BMI, body mass index. Unless otherwise indicated, values are presented as means. ^a^ Values were reported as medians in the original study. N/A indicates that the variable was not reported in the original study.

Intervention protocols varied across the included trials. Four studies used frozen fecal material, and one study used fresh fecal material. With regard to the route of administration, two studies used colonoscopy, one used a nasojejunal tube, and two used oral capsules. In addition, three studies adopted a single-dose FMT regimen, whereas two studies used repeated-dose administration. A summary of these differences in FMT intervention protocols is provided in Table 3 (Ref. [21,22,23,24,25]).

**Table 3.** **Summary of FMT intervention differences**.

Study	Fecal type	Administration route	Dosing
Bruggeman et al., 2024 [21]	Frozen	Nasojejunal	Single
Scheperjans et al., 2024 [22]	Frozen	Colonoscopy	Single
Liu et al., 2023 [23]	Frozen	Oral capsules	Repeated
Wang et al., 2025 [24]	Fresh	Colonoscopy	Single
Cheng et al., 2023 [25]	Frozen	Oral capsules	Repeated

### 3.3 Bias Assessment

**Fig. 2.**
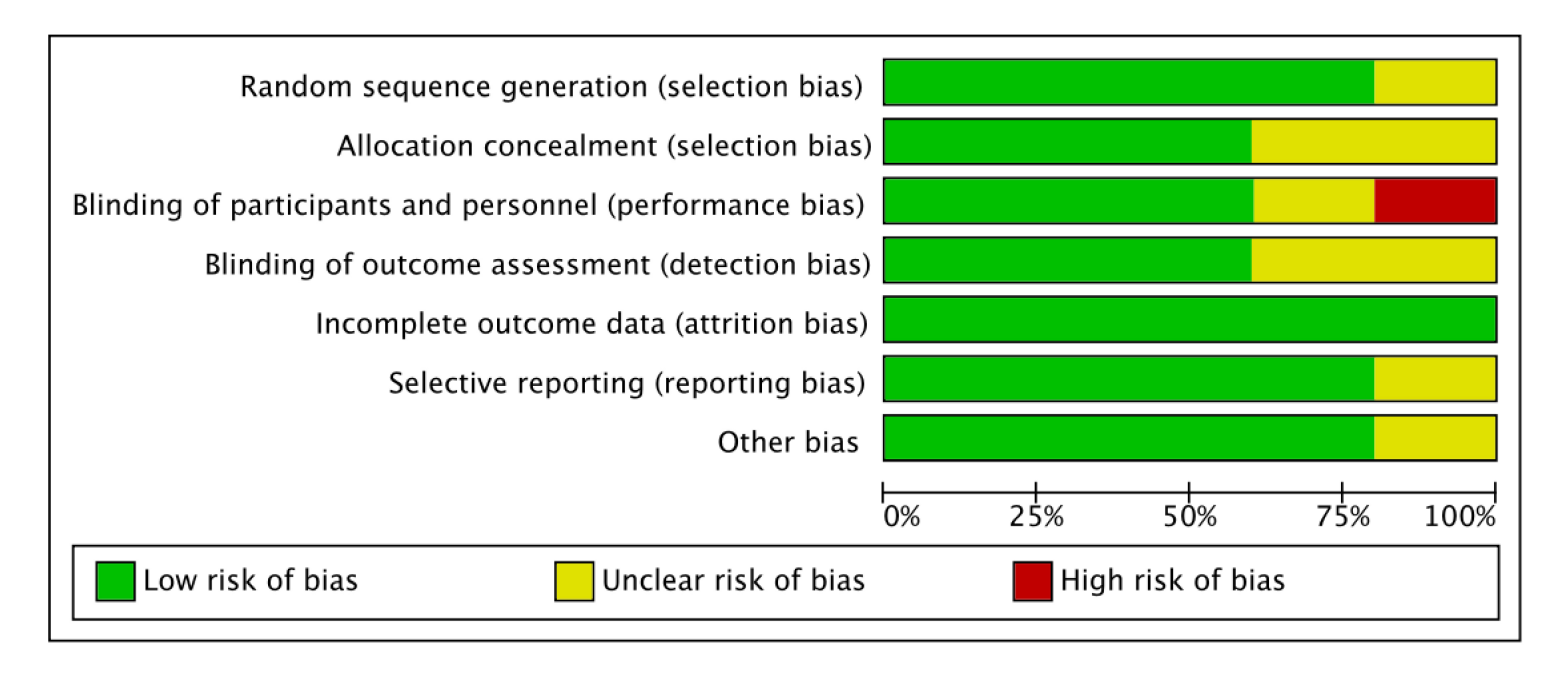
**Review authors’ judgments about each risk of bias item, presented as percentage across all included studies**.

**Fig. 3.**
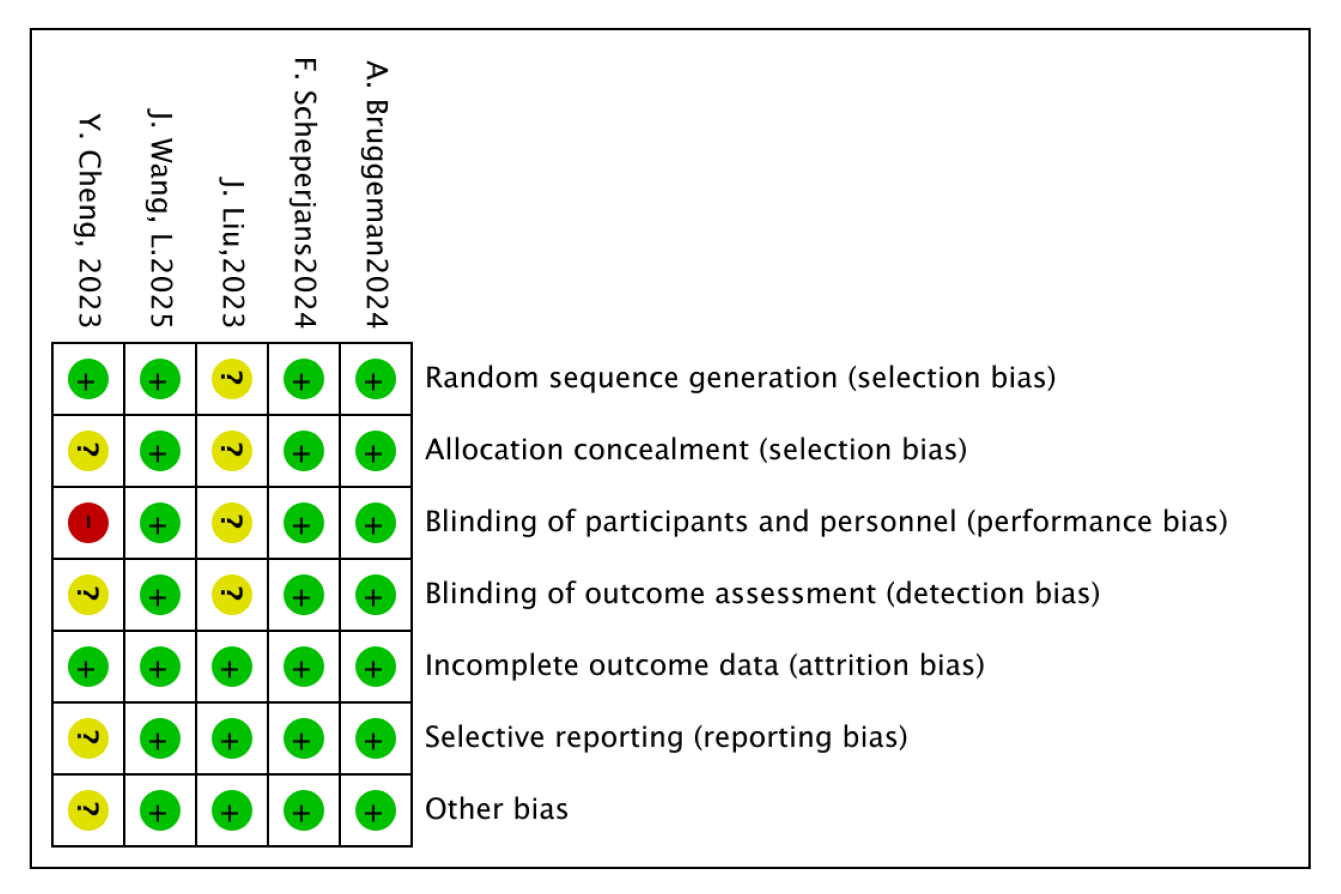
**Risk of bias traffic light plot**. In the risk-of-bias assessment, “+” indicates low risk of bias, “−” indicates high risk of bias, and “?” indicates unclear risk of bias due to insufficient information.

Risk of bias was assessed using the Cochrane Risk of Bias tool for randomized controlled trials [26]. Overall, the methodological quality of the included studies was mixed. One trial was judged to be at high risk of bias, mainly because blinding of participants and study personnel was not clearly implemented. The remaining studies showed some concerns in domains related to blinding and allocation concealment. A summary of the risk of bias assessment is presented in Figs. 2,3.

### 3.4 Analysis of Primary Outcome

#### 3.4.1 Motor and Daily Living Outcomes

**Fig. 4.**
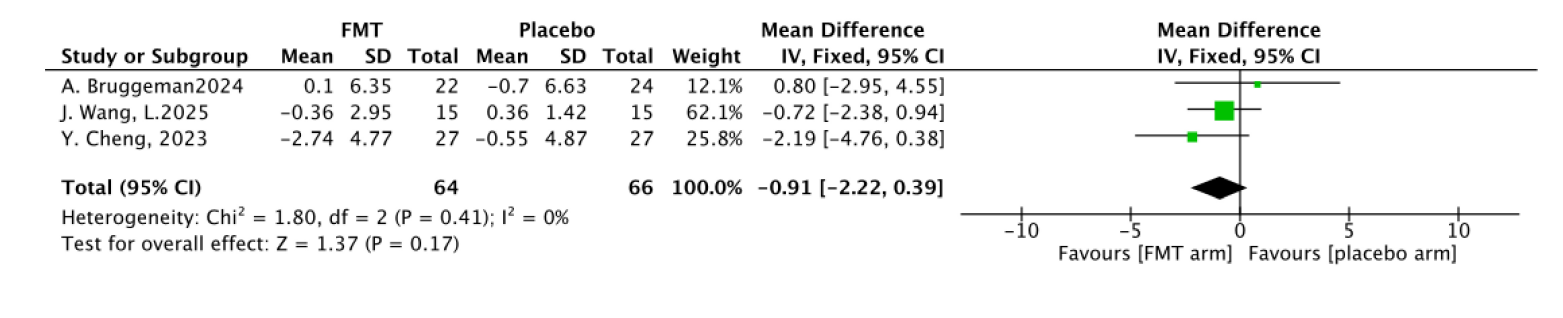
**Forest plot for change in Movement Disorder Society Unified Parkinson’s Disease Rating Scale (MDS-UPDRS) Part I at week 12**.

**Fig. 5.**
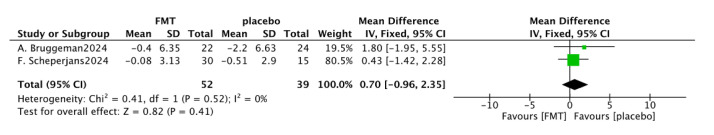
**Forest plot for change in Movement Disorder Society Unified Parkinson’s Disease Rating Scale (MDS-UPDRS) Part I at week 24**.

**Fig. 6.**
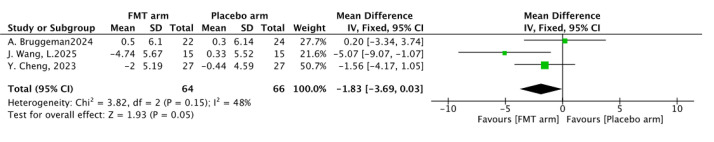
**Forest plot for change in Movement Disorder Society Unified Parkinson’s Disease Rating Scale (MDS-UPDRS) Part Ⅱ at week 12**.

**Fig. 7.**
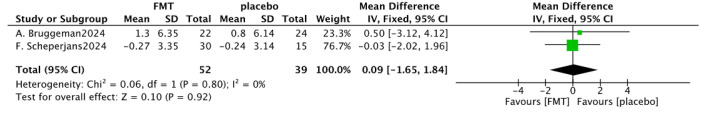
**Forest plot for change in Movement Disorder Society Unified Parkinson’s Disease Rating Scale (MDS-UPDRS) Part Ⅱ at week 24**.

**Fig. 8.**
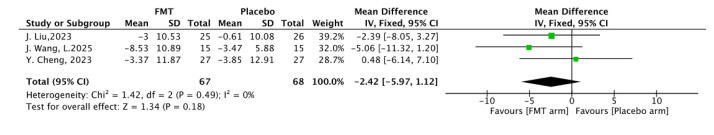
**Forest plot for change in Movement Disorder Society Unified Parkinson’s Disease Rating Scale (MDS-UPDRS) Part Ⅲ at week 4**.

**Fig. 9.**
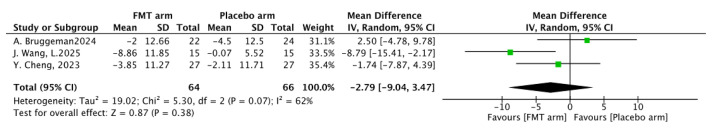
**Forest plot for change in Movement Disorder Society Unified Parkinson’s Disease Rating Scale (MDS-UPDRS) Part Ⅲ at week 12**.

**Fig. 10.**
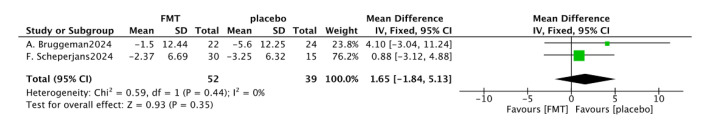
**Forest plot for change in Movement Disorder Society Unified Parkinson’s Disease Rating Scale (MDS-UPDRS) Part Ⅲ at week 24**.

Across the included studies, FMT did not produce statistically significant improvements in MDS-UPDRS Parts I–III at any assessed follow-up time point. For MDS-UPDRS Part I, no significant differences were observed at week 12 (MD = –0.91; 95% CI: –2.22 to 0.39; *p* = 0.17; I^2^ = 0%) or week 24 (MD = 0.70; 95% CI: –0.96 to 2.35; *p* = 0.41; I^2^ = 0%). Similarly, MDS-UPDRS Part II showed no statistically significant difference at week 12 (MD = –1.83; 95% CI: –3.69 to 0.03; *p* = 0.05; I^2^ = 48%) or week 24 (MD = 0.09; 95% CI: –1.65 to 1.84; *p* = 0.92; I^2^ = 0%), although the week-12 result showed a borderline trend favoring FMT. For MDS-UPDRS Part III, no significant effects were detected at week 4 (MD = –2.42; 95% CI: –5.97 to 1.12; *p* = 0.18; I^2^ = 0%), week 12 (MD = –2.79; 95% CI: –9.04 to 3.47; *p* = 0.38; I^2^ = 62%), or week 24 (MD = 1.65; 95% CI: –1.84 to 5.13; *p* = 0.35; I^2^ = 0%) (Figs. 4,5,6,7,8,9,10 and Table 1).

#### 3.4.2 Cognitive Outcomes

**Fig. 11.**
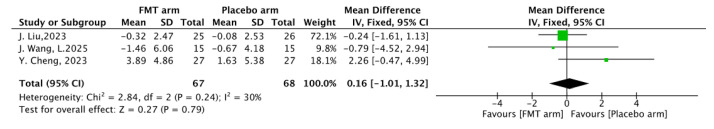
**Forest plot for change in Montreal Cognitive Assessment scores at week 4**.

**Fig. 12.**
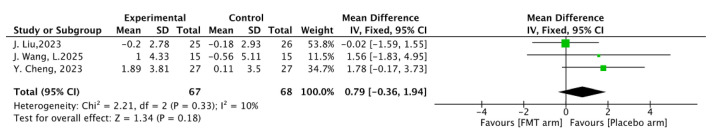
**Forest plot for change in Mini-Mental State Examination scores at week 4**.

Cognitive outcomes were assessed using MoCA and MMSE scores at week 4. No statistically significant difference was observed between the FMT and control groups in MoCA scores (MD = 0.16; 95% CI: –1.01 to 1.32; *p* = 0.79; I^2^ = 30%) or MMSE scores (MD = 0.79; 95% CI: –0.36 to 1.94; *p* = 0.18; I^2^ = 10%) (Figs. 11,12 and Table 1).

### 3.5 Publication Bias and Sensitivity Analysis

Publication bias was explored using funnel plots. However, because each meta-analysis included fewer than 10 studies, funnel-plot symmetry was of limited interpretability, and publication bias could not be reliably assessed.

Sensitivity analysis was conducted to explore the source of heterogeneity. For MDS-UPDRS Part III at week 12, the initial heterogeneity was moderate (I^2^ = 62%). While the leave-one-out sensitivity analysis identified Wang et al. (2025) [24] as the primary source of heterogeneity, this heterogeneity should be interpreted through a clinical lens rather than a purely statistical one. Notably, the Wang et al. [24] study utilized fresh fecal material administered via colonoscopy, whereas other included trials primarily employed frozen preparations. This suggests that the physiological impact of FMT may be highly dependent on bacterial viability and the specific route of administration, necessitating further subgroup analyses in future large-scale trials.

## 4. Discussion

### 4.1 Current Status of FMT RCTs for Parkinson’s Disease

To date, only five RCTs have examined the effects of FMT in PD, reflecting the early stage of clinical research. Across all assessed outcomes (motor function, daily living activities, and cognition), no statistically significant benefit of FMT was detected. A non-significant trend favoring FMT was observed for MDS-UPDRS Part II at week 12, but this was not sustained at later follow-up. These null findings, however, did not necessarily indicate a true absence of effect. The current evidence base remained constrained by small sample sizes and follow-up periods that may be insufficient to capture meaningful clinical change. Differences in background PD medications, together with the rater-dependent nature of scales such as the MDS-UPDRS, may also have contributed to between-study variability.

### 4.2 FMT and Non-Motor Symptoms

Non-motor symptoms in PD, including mood disturbance, sleep impairment, and autonomic dysfunction, are thought to be closely related to gut-brain axis dysregulation [27]. In the present analysis, no significant benefit of FMT was observed for MDS-UPDRS Part I outcomes. Although Wang et al.[24] reported increased abundance of beneficial gut bacteria and reduction of constipation after fresh FMT was administered by colonoscopy, corresponding improvement in other non-motor domains, such as mood or sleep, was not clearly demonstrated. One possible explanation is that a single FMT session may have been insufficient to achieve sustained microbiota engraftment, let alone influence gut barrier integrity or systemic inflammatory signaling. In addition, differences in fecal preparation may also be relevant. For example, Bruggeman et al [21]. and Scheperjans et al [22]. used frozen fecal material, which may have influenced bacterial viability [28]. Because short-chain fatty acids (SCFAs) are considered important mediators of gut-brain communication [29], variation in microbiota composition and metabolite production after FMT may partly have contributed to differences in clinical response. However, those mechanisms were not directly assessed in the included trials and should therefore be interpreted as possible explanations rather than confirmed conclusions. The persistently non-significant findings at 24 weeks indicated that the current evidence remained insufficient to demonstrate a durable benefit of FMT for non-motor symptoms in PD.

### 4.3 FMT and Motor Symptoms

In the present meta-analysis, FMT did not produce a significant improvement in motor outcomes at any assessed time point. Moderate heterogeneity was observed for MDS-UPDRS Part III at week 12 (I^2^ = 62%), and sensitivity analysis suggested that the study by Wang et al. [24] was the primary source of heterogeneity. Although preclinical studies have shown that FMT can improve motor deficits in PD animal models [30], clinical translation remains challenging, and the underlying mechanisms were not directly evaluated in the included trials. These findings may have reflected several factors. First, the pathophysiology of PD motor symptoms is complex and progressive, which may limit the extent to which a microbiota-based intervention can produce measurable short-term motor benefit. Second, differences in FMT protocols across studies, including the use of both fresh and frozen fecal material, may have influenced treatment effects. Frozen preparations in particular may have affected bacterial viability, including bacteria involved in SCFA production [28,31]. Because SCFAs are considered important mediators of gut-brain communication, variation in microbiota composition and metabolite production after FMT may have partly contributed to differences in motor response. These mechanistic explanations remain speculative in the absence of direct mechanistic data from the included trials. Background anti-Parkinson medications, including levodopa-based therapies, may also have influenced motor assessments and contributed to between-study variability.

The analysis of MDS-UPDRS Part II scores at 12 weeks yielded a result at the threshold of statistical significance (MD = –1.83; *p* = 0.05). This suggests a promising trend toward improvement in activities of daily living that may have failed to reach conventional significance levels due to the limited aggregate sample size (n = 130). Consequently, this outcome warrants cautious optimism and highlights the need for adequately powered RCTs to confirm whether FMT provides a meaningful benefit for activities of daily living in patients with PD.

### 4.4 FMT and Cognitive Function

In the present meta-analysis, FMT was not associated with significant improvement in either MoCA or MMSE scores. Cognitive impairment in PD is multifactorial and has been linked to Lewy body pathology, cholinergic dysfunction, and alterations in large-scale brain networks [32,33]. These mechanisms may help explain why a microbiota-based intervention alone did not translate into measurable short-term improvement in cognitive scores in the included trials. Although FMT may modulate gut-microbiota composition and related metabolites, its downstream effects on central cognitive pathways remain uncertain. SCFAs have been proposed as a potential pathway underlying the neuroprotective effects of FMT and have been implicated in gut barrier function, immune regulation, and gut-brain communication [34]. However, SCFAs and other relevant metabolites were not directly quantified in the included trials. Therefore, the mechanistic link between microbial engraftment and cognitive outcomes in PD remains speculative. Future longitudinal multi-omics studies, including fecal and systemic metabolomics, are needed to clarify the biological mediators of the gut-brain axis in PD. Moreover, the current clinical evidence remains limited. No consistent cognitive benefit of FMT has been demonstrated in PD, and evidence from other neurodegenerative conditions, such as Alzheimer’s disease, remains similarly inconclusive [35]. In the included trials, Cheng et al [25]. also found no significant improvement in cognition-related outcomes despite changes in gut microbiota composition, which corresponded with the pooled findings of the present meta-analysis.

## 5. Limitations

This study had several limitations. First, only five randomized controlled trials were included, and the total sample size was small, which limited statistical power and reduced the ability to detect modest treatment effects. Second, there was notable clinical heterogeneity across studies, including differences in fecal preparation (fresh vs. frozen), route of administration, dosing frequency, and follow-up duration. Although statistical heterogeneity was low for several outcomes, such protocol differences may still have affected the comparability of results across studies. In addition, variability in outcome measurement and adverse event definitions may have further limited cross-study comparability. Third, because fewer than 10 studies were available for each meta-analysis, funnel plots were of limited interpretability, and publication bias could not be reliably assessed. Finally, the interpretability of the pooled estimates is subject to several methodological constraints. First, the necessity of converting median values to means in 40% of the included studies introduces inherent statistical uncertainty. Second, the rater-dependent nature of the MDS-UPDRS scale, combined with ‘unclear’ or ‘high’ risk of bias in participant and personnel blinding in certain trials, may have introduced performance or detection bias. These factors emphasize the importance of adopting objective digital biomarkers in future FMT clinical trials.

## 6. Conclusions

In conclusion, while current randomized evidence does not support a statistically significant benefit of FMT for motor or cognitive outcomes in Parkinson’s disease, the safety profile and encouraging trends in daily living scores justify continued investigation. Rather than a definitive rejection of FMT, these null findings likely reflect the preliminary nature of the field, characterized by heterogeneous protocols and insufficient follow-up durations.

## Data Availability

The data that support the findings of this study are available from the corresponding author upon reasonable request. All data utilized in this meta-analysis were extracted from previously published research articles listed in the reference section.
