# Supplementary material for: Systematic Review and Meta-Analysis of the Efficacy of Fecal Microbiota Transplantation in Parkinson’s Disease: An Exploration Based on UPDRS and Cognitive Scores
Source: Rev Neurol. 2026 Jun 26;81(6):50106. doi: 10.31083/RN50106 (PMC13339778; doi:10.31083/RN50106)
Supplement: Supplementary file 1 [file 1576-6578-81-6-50106-s1.zip › Supplementary Material.docx]

# PRISMA 2020 Main Checklist

| **Topic** | **No.** | **Item** | **Location where item is reported** |
| --- | --- | --- | --- |
| **TITLE** |  |  |  |
| **Title** | 1 | Identify the report as a systematic review. | Manuscript title: Systematic Review and Meta-Analysis of the Efficacy of Fecal Microbiota Transplantation in Parkinson’s Disease: An Exploration Based on UPDRS and Cognitive Scores, explicitly labelled as a systematic review and meta-analysis |
| **ABSTRACT** |  |  |  |
| **Abstract** | 2 | See the PRISMA 2020 for Abstracts checklist | The full abstract strictly complies with PRISMA abstract standards, covering background, methods, results, conclusions and PROSPERO registration information |
| **INTRODUCTION** |  |  |  |
| **Rationale** | 3 | Describe the rationale for the review in the context of existing knowledge. | Paragraph 2 of Introduction: Elaborates gut-brain axis mechanisms and evidence of gut microbiota dysbiosis in Parkinson’s disease (PD); clarifies the theoretical potential of FMT yet insufficient and inconsistent RCT evidence, demonstrating the necessity of this meta-analysis |
| **Objectives** | 4 | Provide an explicit statement of the objective(s) or question(s) the review addresses. | Final paragraph of Introduction: To systematically evaluate the effects of fecal microbiota transplantation (FMT) on motor, non-motor and cognitive outcomes in patients with PD |
| **METHODS** |  |  |  |
| **Eligibility criteria** | 5 | Specify the inclusion and exclusion criteria for the review and how studies were grouped for the syntheses. | Section 2.2 Eligibility Criteria: PICOS framework-based inclusion/exclusion criteria; subgroup meta-analyses stratified by follow-up time points (week 4 / week 12 / week 24) and outcome types (MDS-UPDRS Part I/II/III, MoCA, MMSE) |
| **Information sources** | 6 | Specify all databases, registers, websites, organisations, reference lists and other sources searched or consulted to identify studies. Specify the date when each source was last searched or consulted. | Section 2.1 Data Sources and Search Strategy: Databases including PubMed, Ovid Embase, Scopus and Cochrane CENTRAL; search deadline: 15 September 2025; manual screening of reference lists of included articles and relevant reviews; ClinicalTrials.gov was not searched |
| **Search strategy** | 7 | Present the full search strategies for all databases, registers and websites, including any filters and limits used. | Section 2.1 states database-specific adapted search syntax; full search strings are provided in this supplementary material |
| **Selection process** | 8 | Specify the methods used to decide whether a study met the inclusion criteria of the review, including how many reviewers screened each record and each report retrieved, whether they worked independently, and if applicable, details of automation tools used in the process. | Section 2.3 Study Selection: All citations imported to EndNote 21 for duplicate removal; two independent reviewers screened titles/abstracts then full texts; disagreements resolved via discussion or consultation with a third reviewer; selection workflow visualized in PRISMA flow diagram (Figure 1) |
| **Data collection process** | 9 | Specify the methods used to collect data from reports, including how many reviewers collected data from each report, whether they worked independently, any processes for obtaining or confirming data from study investigators, and if applicable, details of automation tools used in the process. | Section 2.4 Data Extraction and Quality Assessment: Two independent reviewers extracted data via standardized Microsoft Excel forms; discrepancies adjudicated by a third reviewer; no contact with original trial authors for unpublished raw data |
| **Data items** | 10a | List and define all outcomes for which data were sought. Specify whether all results that were compatible with each outcome domain in each study were sought (e.g. for all measures, time points, analyses), and if not, the methods used to decide which results to collect. | Section 2.4: Primary outcomes = changes in MDS-UPDRS Part I/II/III at predefined follow-ups; secondary outcomes = MoCA and MMSE cognitive score changes; all quantifiable data at week 4/12/24 were extracted) |
|  | 10b | List and define all other variables for which data were sought (e.g. participant and intervention characteristics, funding sources). Describe any assumptions made about any missing or unclear information. | Extracted covariates: study location, sample size, baseline age/BMI/PD duration, FMT protocols (fecal material type, administration route, dosing frequency), control regimens, follow-up length; missing SD of change calculated with correlation coefficient r=0.3/0.5/0.7; medians converted to means via Wan’s method |
| **Study risk of bias assessment** | 11 | Specify the methods used to assess risk of bias in the included studies, including details of the tool(s) used, how many reviewers assessed each study and whether they worked independently, and if applicable, details of automation tools used in the process. | Section 2.4: Cochrane Risk of Bias Tool for RCTs adopted; two independent raters evaluated random sequence generation, allocation concealment, blinding, incomplete outcome data and selective reporting; disagreements resolved by a third reviewer; results summarized in Figure 2 and Figure 3 |
| **Effect measures** | 12 | Specify for each outcome the effect measure(s) (e.g. risk ratio, mean difference) used in the synthesis or presentation of results. | Section 2.5 Data Synthesis and Statistical Analysis: Mean difference (MD) with 95% confidence intervals (CIs) as pooled effect size for continuous outcomes |
| **Synthesis methods** | 13a | Describe the processes used to decide which studies were eligible for each synthesis (e.g. tabulating the study intervention characteristics and comparing against the planned groups for each synthesis (item 5)). | Only RCTs fulfilling PICOS criteria were pooled; subgroup meta-analyses grouped by outcome indicator and follow-up time; characteristics of included trials summarized in Table 2 and Table 3 |
|  | 13b | Describe any methods required to prepare the data for presentation or synthesis, such as handling of missing summary statistics, or data conversions. | Missing SD of change computed via dedicated formula; median-to-mean transformation applied where necessary; sensitivity analyses with r=0.3/0.5/0.7 to test result robustness |
|  | 13c | Describe any methods used to tabulate or visually display results of individual studies and syntheses. | Baseline participant characteristics (Table 2), FMT intervention profiles (Table 3), GRADE evidence certainty summary (Table 1); pooled effects presented via forest plots (Figure 4–Figure 12); risk of bias summary plot (Figure 2) and traffic light plot (Figure 3) |
|  | 13d | Describe any methods used to synthesize results and provide a rationale for the choice(s). If meta-analysis was performed, describe the model(s), method(s) to identify the presence and extent of statistical heterogeneity, and software package(s) used. | RevMan 5.4.1 applied; I² statistic to quantify heterogeneity; fixed-effect model for I² ≤ 50%, random-effects model for I² > 50%; leave-one-out sensitivity analysis to explore sources of heterogeneity |
|  | 13e | Describe any methods used to explore possible causes of heterogeneity among study results (e.g. subgroup analysis, meta-regression). | Sensitivity analysis identified heterogeneity drivers; inconsistency attributed to divergent FMT protocols (fresh vs frozen stool, delivery routes); subgroup analyses recommended for future large-scale trials |
|  | 13f | Describe any sensitivity analyses conducted to assess robustness of the synthesized results. | Two types of sensitivity analyses: 1) varying correlation coefficient r (0.3 / 0.5 / 0.7); 2) leave-one-out analysis confirming Wang et al. (2025) as the primary source of heterogeneity for MDS-UPDRS Part III at week 12 |
| **Reporting bias assessment** | 14 | Describe any methods used to assess risk of bias due to missing results in a synthesis (arising from reporting biases). | Section 3.5 Publication Bias and Sensitivity Analysis: Funnel plots generated to assess publication bias; however, each meta-analysis contained fewer than 10 studies, limiting interpretability and preventing reliable evaluation of publication bias |
| **Certainty assessment** | 15 | Describe any methods used to assess certainty (or confidence) in the body of evidence for an outcome. | Section 2.4: Two independent reviewers rated evidence certainty via GRADE framework; downgrade factors included high risk of bias, inconsistency, wide CIs and imprecision; GRADE ratings for all outcomes listed in Table 1 |
| **RESULTS** |  |  |  |
| **Study selection** | 16a | Describe the results of the search and selection process, from the number of records identified in the search to the number of studies included in the review, ideally using a flow diagram. | Section 3.1 Literature Selection: Initial search retrieved 109 records; 40 duplicates removed, 56 excluded after title/abstract screening, 13 assessed for full-text eligibility; finally 5 RCTs with 226 participants included; selection workflow illustrated in PRISMA flow diagram (Figure 1) |
|  | 16b | Cite studies that might appear to meet the inclusion criteria, but which were excluded, and explain why they were excluded. | Section 3.1 and Figure 1: Full-text exclusions mainly due to lack of quantitative target outcomes, non-RCT design, case series, reviews or conference abstracts |
| **Study characteristics** | 17 | Cite each included study and present its characteristics. | Section 3.2 Study Characteristics: 5 RCTs published 2023–2025 (1 Belgium, 1 Finland, 3 China); total sample n=226 (FMT=119, control=107); demographic baseline and intervention details summarized in Table 2 & Table 3, references [21–25] cited |
| **Risk of bias in studies** | 18 | Present assessments of risk of bias for each included study. | Section 3.3 Bias Assessment: Mixed methodological quality across trials; one trial rated high risk of bias due to unclear blinding of participants and personnel; remaining studies raised concerns regarding blinding and allocation concealment; visualized in Figure 2 (summary plot) and Figure 3 (traffic light plot) |
| **Results of individual studies** | 19 | For all outcomes, present, for each study: (a) summary statistics for each group (where appropriate) and (b) an effect estimate and its precision (e.g. confidence/credible interval), ideally using structured tables or plots. | Table 1 reports MD and 95% CI for each outcome; forest plots (Figure 4–12) display individual study mean, SD, weight and pooled effects; baseline demographic and intervention data in Table 2 & Table 3 |
| **Results of syntheses** | 20a | For each synthesis, briefly summarise the characteristics and risk of bias among contributing studies. | Section 3.4 briefly describes number of trials, total sample size, overall risk of bias and heterogeneity level prior to each subgroup meta-analysis |
|  | 20b | Present results of all statistical syntheses conducted. If meta-analysis was done, present for each the summary estimate and its precision (e.g. confidence/credible interval) and measures of statistical heterogeneity. If comparing groups, describe the direction of the effect. | Section 3.4.1 Motor/Activities of Daily Living outcomes & 3.4.2 Cognitive outcomes report all pooled MD, 95% CI, p-value and I²; all CIs crossed zero with no statistically significant between-group difference; only MDS-UPDRS Part II at week 12 showed borderline trend favouring FMT (p=0.05) |
|  | 20c | Present results of all investigations of possible causes of heterogeneity among study results. | Section 3.5: I²=62% for MDS-UPDRS Part III week 12; leave-one-out sensitivity analysis identified Wang et al. (2025) (fresh stool administered via colonoscopy) as the main source of heterogeneity, driven by divergent FMT preparation and delivery routes |
|  | 20d | Present results of all sensitivity analyses conducted to assess the robustness of the synthesized results. | Section 3.5 fully describes two sensitivity approaches: varying correlation coefficients and leave-one-out exclusion; pooled conclusions remained materially unchanged, confirming robust results |
| **Reporting biases** | 21 | Present assessments of risk of bias due to missing results (arising from reporting biases) for each synthesis assessed. | Section 3.5: Funnel plots attempted but underpowered due to limited study count; reliable publication bias evaluation impossible, limitation discussed in Section 5 Limitations |
| **Certainty of evidence** | 22 | Present assessments of certainty (or confidence) in the body of evidence for each outcome assessed. | Table 1 GRADE summary table: Most outcomes rated moderate certainty; MDS-UPDRS Part III week 12 downgraded to low certainty due to substantial heterogeneity; downgrade reasons = wide confidence intervals + high inconsistency for selected outcomes |
| **DISCUSSION** |  |  |  |
| **Discussion** | 23a | Provide a general interpretation of the results in the context of other evidence. | Section 4.1–4.4: Compare pooled null RCT findings with positive preclinical animal FMT data and small observational clinical series; interpret results via gut-brain axis, short-chain fatty acid and microbiota engraftment mechanisms |
|  | 23b | Discuss any limitations of the evidence included in the review. | Section 5 Limitations: Small number of included trials and limited total sample size; high clinical heterogeneity across FMT protocols (fresh/frozen stool, delivery routes, dosing frequency); short follow-up duration; statistical bias introduced by median-to-mean conversion; unclear/high blinding risk in several trials |
|  | 23c | Discuss any limitations of the review processes used. | Section 5: ClinicalTrials.gov not searched; fewer than 10 studies per meta-analysis preventing formal publication bias assessment; only peer-reviewed published articles included, potential language bias) |
|  | 23d | Discuss implications of the results for practice, policy, and future research. | Section 6 Conclusions: Current RCT evidence does not support statistically significant improvements in motor or cognitive function via FMT; borderline beneficial trend for activities of daily living at week 12 warrants further validation; large-scale multi-center RCTs with standardized FMT protocols and prolonged follow-up are required; multi-omics metabolomics assays recommended to explore underlying biological mechanisms |
| **OTHER INFORMATION** |  |  |  |
| **Registration and protocol** | 24a | Provide registration information for the review, including register name and registration number, or state that the review was not registered. | Abstract, main text and end declarations: This systematic review was prospectively registered on PROSPERO, registration number CRD420251121443, registration URL: https://www.crd.york.ac.uk/PROSPERO/view/CRD420251121443 |
|  | 24b | Indicate where the review protocol can be accessed, or state that a protocol was not prepared. | Full review protocol accessible via the PROSPERO registry using the above registration ID |
|  | 24c | Describe and explain any amendments to information provided at registration or in the protocol. | No amendments or modifications made to the registered PROSPERO protocol throughout the review |
| **Support** | 25 | Describe sources of financial or non-financial support for the review, and the role of the funders or sponsors in the review. | Acknowledgments section: No external funding received for this research; funders had no role in study design, data extraction, statistical analysis or manuscript drafting |
| **Competing interests** | 26 | Declare any competing interests of review authors. | Conflicts of Interest section: All authors declare no conflicts of interest |
| **Availability of data, code and other materials** | 27 | Report which of the following are publicly available and where they can be found: template data collection forms; data extracted from included studies; data used for all analyses; analytic code; any other materials used in the review. | Availability of Data and Materials section: Extracted datasets available upon reasonable request to the corresponding author; all raw source data extracted from published cited references |

#####

# PRIMSA Abstract Checklist

| **Topic** | **No.** | **Item** | **Reported?** |
| --- | --- | --- | --- |
| **TITLE** |  |  |  |
| **Title** | 1 | Identify the report as a systematic review. | Yes |
| **BACKGROUND** |  |  |  |
| **Objectives** | 2 | Provide an explicit statement of the main objective(s) or question(s) the review addresses. | Yes |
| **METHODS** |  |  |  |
| **Eligibility criteria** | 3 | Specify the inclusion and exclusion criteria for the review. | Yes |
| **Information sources** | 4 | Specify the information sources (e.g. databases, registers) used to identify studies and the date when each was last searched. | Yes |
| **Risk of bias** | 5 | Specify the methods used to assess risk of bias in the included studies. | Yes |
| **Synthesis of results** | 6 | Specify the methods used to present and synthesize results. | Yes |
| **RESULTS** |  |  |  |
| **Included studies** | 7 | Give the total number of included studies and participants and summarise relevant characteristics of studies. | Yes |
| **Synthesis of results** | 8 | Present results for main outcomes, preferably indicating the number of included studies and participants for each. If meta-analysis was done, report the summary estimate and confidence/credible interval. If comparing groups, indicate the direction of the effect (i.e. which group is favoured). | Yes |
| **DISCUSSION** |  |  |  |
| **Limitations of evidence** | 9 | Provide a brief summary of the limitations of the evidence included in the review (e.g. study risk of bias, inconsistency and imprecision). | Yes |
| **Interpretation** | 10 | Provide a general interpretation of the results and important implications. | Yes |
| **OTHER** |  |  |  |
| **Funding** | 11 | Specify the primary source of funding for the review. | Yes |
| **Registration** | 12 | Provide the register name and registration number. | Yes |

*From:* Page MJ, McKenzie JE, Bossuyt PM, Boutron I, Hoffmann TC, Mulrow CD, et al. The PRISMA 2020 statement: an updated guideline for reporting systematic reviews. MetaArXiv. 2020, September 14. DOI: 10.31222/osf.io/v7gm2. For more information, visit: <www.prisma-statement.org>
